# Validity of magnetic resonance imaging (MRI) in the primary spinal cord tumors in routine clinical setting

**DOI:** 10.1038/s41598-022-13881-z

**Published:** 2022-06-16

**Authors:** Young Il Won, Yunhee Choi, Woon Tak Yuh, Shin Won Kwon, Chi Heon Kim, Seung Heon Yang, Chun Kee Chung

**Affiliations:** 1 Department of Neurosurgery, Seoul National University Hospital, Seoul, Republic of Korea; 2 Division of Medical Statistics, Medical Research Collaborating Center, Seoul National University Hospital, Seoul, Republic of Korea; 3 Department of Neurosurgery, Seoul National University College of Medicine, 101 Daehak ro, Jongno-gu, Seoul, 03080 Republic of Korea; 4 Department of Brain and Cognitive Sciences, Seoul National University, Seoul, Republic of Korea

**Keywords:** Surgical oncology, Spinal cord diseases

## Abstract

MRI is the primary diagnostic modality for spinal cord tumors. However, its validity has never been vigorously scrutinized in daily routine clinical practice, where MRI tissue diagnosis is usually not a single one but multiple ones with several differential diagnoses. Here, we aimed to assess the validity of MRI in terms of predicting the pathology and location of the tumor in routine clinical settings. We analyzed 820 patients with primary spinal cord tumors, who have a pathological diagnosis and location in the operation record which were confirmed. We modified traditional measures for validity based upon a set of diagnoses instead of a single diagnosis. Sensitivity and specificity and positive and negative predictabilities were evaluated for the tumor location and pathology. For tumor location, 456 were intradural extramedullary; 165 were intramedullary, and 156 were extradural. The overall sensitivity and specificity were over 90.0%. However, the sensitivity became lower when the tumor resided simultaneously in two spaces such as in the intradural-and-extradural or intramedullary-and-extramedullary space (54.6% and 30.0%, respectively). Most common pathology was schwannoma (n = 416), followed by meningioma (114) and ependymoma (87). Sensitivities were 93.3%, 90.4%, and 89.7%, respectively. Specificities were 70.8%, 82.9%, and 76.0%. In rare tumors such as neurofibromas, and diffuse midline gliomas, the sensitivity was much lower (less than 30%). For common locations and pathologies, the validity of MRI is generally acceptable. However, for rare locations and pathologies, MRI diagnosis still needs some improvement.

## Introduction

Primary spinal cord tumors constitute 2% to 4% of all central nervous system tumors. The location of the primary spinal cord tumors is usually categorized into 3 locations: intramedullary (IM), intradural extramedullary (IDEM), and extradural (ED)^1,2^. One of the major impetuses that have led remarkable advancements in the field of primary spinal cord tumor surgery was the introduction of MRI^3–5^. Precise prediction of both the pathology and location of a tumor helps presurgical planning and counseling with improved surgical outcomes^3^. However, limited studies have been published with regards to the validity of MRI in primary spinal cord tumors. There have been only a few studies with a limited number of cases that have investigated the validity MRI in primary spinal cord tumors^3,6–8^. They did not consider the location of the tumor. Additionally, MRI tissue diagnosis is usually not a single but multiple with several plausible differential diagnoses. Hence, the traditional measures for validity could not reflect the situation in daily routine clinical practice.

In the present study, we aimed to assess the validity of MRI for both the location and pathology of primary spinal cord tumors reflecting daily routine clinical practice. Second, we explored whether there is variability in the validity by each neuroradiologist and by the periods.

## Methods

### Patient population

From January 2003 to March 2019, the pathological results and operative records of 1173 patients were retrospectively reviewed who were treated at Seoul National University Hospital (SNUH) and diagnosed with a primary spinal cord lesion. Excluded were patients with primary bone tumors such as chordoma or giant cell tumors, metastatic tumors, or extraforaminal tumors. Revision cases and patients with preoperative MRI not available were excluded as well. Based on the exclusion criteria, finally, a total of 820 patients were analyzed. This study was conducted in accordance with the Declaration of Helsinki and the Guideline for Good Clinical Practice. All data were recorded prospectively using an electronic medical recording system (IRB No. 0507-509-153). The present study was approved by the Seoul National University Hospital ethics committee/institutional review board (IRB No. 2111-197-1279) and was exempted from informed consent requirements owing to its retrospective design.

### Data management

The location of primary spinal cord tumors was decided based upon the operation records. It was determined whether the dura or pia mater was opened. Pathological specimens obtained during surgery were examined by neuropathologists. To reflect daily routine clinical practice, we accepted the report of the neuroradiologist as it is rather than re-interpreting MRI scans by a panel of experts. All the neuroradiologists have more than ten years of experience. The location of the tumor by MRI reports and their diagnosis were each compared with the reference standards set as the tumor location identified by operation record and the pathology reported in pathological reports, respectively.

Since MRI tissue diagnosis was multiple, we modified the traditional measures for the validity based upon a set of diagnoses instead of a single diagnosis. For pathology ‘X’, if the set of differential diagnoses include ‘X’, it is counted as a true positive. If pathology is not ‘X’ and the set does not include ‘X’, it is considered as true negative.

Clinically, differentiation of schwannomas from meningiomas and ependymomas from other gliomas is important. We evaluated how well MRI distinguishes these tumors in confusing cases. In addition, the validity of MRI was compared in the first half period and the second half, and for each of the top four neuroradiologists in the number of cases.

### Statistical analysis

Sensitivity, specificity, positive predictive value (PPV), negative predictive value (NPV), and corresponding 95% confidence intervals (CIs) were evaluated for the location and pathology. Interrupted time series analysis and one-way ANOVA were used to evaluate the specificity and sensitivity according to the period and neuroradiologists. Data were analyzed via the SPSS software package version 23.0 (SPSS, Chicago, ILL, USA).

## Results

### Validity to tumor location

Of the 820 patients, 376 (45.9%) were male, and 444 (54.1%) were female. The mean age at the time of surgery was 49.0 ± 15.1 years. Table 1 depicts the recorded tumor location and validity of MRI identification of the tumor location. IDEM tumor was the most common (55.6%), followed by IM (20.1%) and ED (19.0%) tumors. The overall sensitivity of precise MRI identification of tumor location was 90.0%. However, sensitivities for ID&ED and IM&EM were low (54.6% and 30.0%). Validity according to spine level can be found in Supplementary Table S1 online. Sensitivity and specificity for location were found to be different according to spine level (P = 0.029, 0.04). Post hoc analysis revealed that the sensitivity and specificity of the cervical level (86.4, 96.6%) were lower than that of the thoracic level (93.7, 98.4%) (P = 0.034, 0.046). The sensitivity and specificity of the thoracolumbar level were 86.5 and 99.6%, and the lumbar level was 91.2 and 97.8%, which showed no statistically significant difference from other levels.

**Table 1:** The validity of MRI for the location of the tumor.

Reference standard	MRI report						Validity (95% confidence interval)			
	IDEM	IM	ED	ID&ED	IM&EM	Total (%)	Sensitivity	Specificity	PPV	NPV
IDEM	425	17	7	5	2	456 (55.6)	0.932 (0.909, 0.955)	0.92 (0.893, 0.948)	0.936 (0.914, 0.959)	0.915 (0.887, 0.944)
IM	10	154	0	0	1	165 (20.1)	0.933 (0.895, 0.971)	0.97 (0.956, 0.983)	0.885 (0.838, 0.932)	0.983 (0.973, 0.993)
ED	7	1	138	10	0	156 (19.0)	0.885 (0.835, 0.935)	0.977 (0.966, 0.989)	0.902 (0.855, 0.949)	0.973 (0.961, 0.985)
ID&ED^a^	7	0	8	18	0	33 (4.0)	0.546 (0.376, 0.715)	0.981 (0.971, 0.991)	0.546 (0.376, 0.715)	0.981 (0.971, 0.991)
IM&EM	5	2	0	0	3	10 (1.2)	0.3 (0.016, 0.584)	0.996 (0.989, 0.999)	0.5 (0.1, 0.9)	0.991 (0.985, 0.998)
Total						820	0.9 (0.88, 0.921)	0.975 (0.969, 0.980)	0.9 (0.88, 0.921)	0.975 (0.969, 0.980)

*IDEM* inradural extra, *IM* intramedullary, *ED* extradural, *ID&ED* both intradural and extradural, *IM&EM* both intramedullary and extramedullary, *PPV* positive predictive value, *NPV* negative predictive value.

### Pathology

The most common tumor types were schwannoma, meningioma, ependymoma. The frequency of tumor type based on its location is presented in Table 2. The most common IDEM tumors were schwannoma (60.1%) and meningioma (23.7%). Ependymoma (41.2%) and hemangioblastoma (13.3%) were the most common IM tumors. Schwannoma (64.7%) and cavernous malformation (8.3%) were the most common ED tumors. The distribution of tumors by spine level can be found online in Supplementary Table S2. The proportion of schwannoma at cervical and thoracic level (39.1, 37.4%) were lower than at thoracolumbar and lumbar level (62.5, 73.1%) (P < 0.001).

**Table 2:** Frequency of tumors by location.

Tumor type	No. of cases	Percentage	Location				
			IDEM	IM	ED	ID&ED	IM&EM
Schwannoma	416	50.73	274 (60.1%)*	14 (8.5%)	101 (64.7%)*	23 (69.7%)*	4 (40.0%)*
Meningioma	114	13.9	108 (23.7%)*		2 (1.3%)	4 (12.1%)	
Ependymoma	87	10.61	17 (3.7%)	68 (41.2%)*			2 (20.0%)
Hemangioblastoma	28	3.41	5 (1.1%)	22 (13.3%)*			1 (10.0%)
Cavernous malformation	27	3.29	1 (0.2%)	13 (7.9%)	13 (8.3%)*		
Benign cyst	22	2.68	12 (2.6%)	2 (1.2%)	8 (5.1%)		
Neurofibroma	17	2.07	4 (0.9%)		10 (6.4%)	2 (6.1%)	1 (10.0%)
Astrocytoma	13	1.59		13 (7.9%)			
Diffuse midline glioma	10	1.22		10 (6.1%)			
Capillary hemangioma	8	0.98	6 (1.3%)	2 (1.2%)			
Ganglioneuroma	8	0.98	2 (0.4%)		2 (1.3%)	4 (12.1%)	
Hemangiopericytoma	7	0.85	4 (0.9%)		2 (1.3%)		1 (10.0%)
Angiolipoma	5	0.61		1 (0.6%)	4 (2.6%)		
Chordoma	5	0.61			5 (3.2%)		
Dermoid/Epidermoid cyst	4	0.49	3 (0.7%)	1 (0.6%)			
Hematoma	4	0.49	1 (0.2%)	3 (1.8%)			
Malignant peripheral nerve sheath tumor	4	0.49	2 (0.4%)		1 (0.6%)		1 (10.0%)
Glioblastoma	3	0.37	1 (0.2%)	2 (1.2%)			
Inflammation	3	0.37	3 (0.7%)				
Lymphoma	3	0.37		3 (1.8%)			
Myelitis	3	0.37		3 (1.8%)			
Paraganglioma	3	0.37	3 (0.7%)				
Venous malformation	3	0.37		1 (0.6%)	2 (1.3%)		
Lipoma	2	0.24	2 (0.4%)				
Metastasis	2	0.24	1 (0.2%)	1 (0.6%)			
Teratoma	2	0.24	2 (0.4%)				
Glioneuronal tumor	2	0.24		2 (1.2%)			
Abscess	1	0.12			1 (0.6%)		
Alveolar soft part sarcoma	1	0.12			1 (0.6%)		
Benign notochordal cell tumor	1	0.12			1 (0.6%)		
Fibrosclerosis	1	0.12			1 (0.6%)		
Ganglioglioma	1	0.12		1 (0.6%)			
Germinoma	1	0.12		1 (0.6%)			
Inflammatory myofibroblastic tumor	1	0.12	1 (0.2%)				
Leiomyosarcoma	1	0.12	1 (0.2%)				
Multiple myeloma	1	0.12			1 (0.6%)		
Nerve sheath myxoma	1	0.12	1 (0.2%)				
Neurocysticercosis	1	0.12	1 (0.2%)				
Oligodendroglioma	1	0.12		1 (0.6%)			
Sarcoma	1	0.12			1 (0.6%)		
Schwann cell onion bulb tumor	1	0.12	1 (0.2%)				
Vascular malformation	1	0.12		1 (0.6%)			
Total	820		456	165	156	33	10

Asterisk indicates the two most common tumors by location.

The proportion of meningioma at thoracic level (28.4%) was higher than at cervical, thoracolumbar, and lumbar level (11.9, 6.3, 3.1%) (P < 0.001). The proportion of ependymoma at cervical (18.5%) was higher than the other levels (thoracic, 8.7; thoracolumbar, 7.3; lumbar, 5.7%) (P < 0.001). As a result, the overall distribution of tumors was statistically different according to the spine level (P < 0.001).

### Validity of diagnosis

**Figure 1:**
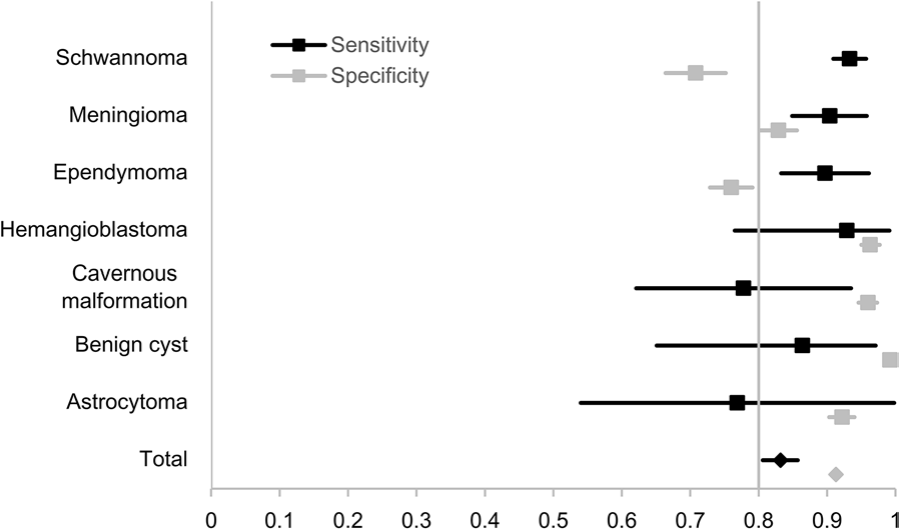
The validity of MRI for pathology.

Sensitivity evaluated by the set of diagnoses was 83.2% (95% CI: 80.6 to 85.7%) for the overall patient population. The overall specificity pooling the top 10 tumors was 91.3% (90.7 to 92.5%). Test validity results of more than 10 cases for each tumor type are presented in Fig. 1. Validities for each tumor type were as follows: schwannoma (sensitivity, 93.3%; specificity, 70.8%), meningioma (90.4%; 82.9%), ependymoma (89.7%; 76.0%), hemangioblastoma (92.9%; 96.3%), cavernous malformation (77.8%; 96.0%), benign cyst (86.4%; 99.6%), astrocytoma (76.9%; 92.2%), neurofibroma (29.4%; 96.0%), diffuse midline glioma (30.0%; 99.9%). In cases with MRI tissue diagnosis was consistent with pathology, 98.5% of cases were predicted within the second impression (Table 3). The sensitivity according to the spine level was statistically different (P = 0.024). However, in post hoc analysis, the sensitivity of cervical and thoracolumbar was 79.4 and 77.1%, respectively, which tended to be lower than that of lumbar (88.1%), but was not statistically significant (P = 0.53, 0.63). The sensitivity of the thoracic level was 84.6% and showed no statistically significant difference from other levels. The specificities of cervical, thoracic, thoracolumbar, and lumbar levels were 92.0, 92.0, 89.1, and 90.9%, respectively. There was no significant difference between the levels. Details can be found online in Supplementary Table S3.

**Table 3:** Consistency of MRI and pathology in each differential diagnosis.

	No. of cases
**True positive**	682
1st differential diagnosis	585 (85.8%)
2nd differential diagnosis	87 (12.8%)
3rd differential diagnosis	7 (1.0%)
4th differential diagnosis	3 (0.4%)
False negative	138
Total	820

### Schwannoma vs. meningioma and ependymoma vs. other glioma

Using 274 IDEM schwannomas and 108 IDEM meningiomas, we evaluated how well MRI differentiated two tumors. An indistinguishable case was defined as a case in which, despite the pathology was schwannoma, meningioma was mentioned in the MRI report, and vice versa. 83 cases of schwannomas (30.3%) and 28 cases of meningiomas (25.9%) were classified as indistinguishable cases. Similarly, 70 cases of IM ependymomas and 25 cases of other gliomas, including astrocytoma, diffuse midline glioma, and glioblastoma, were analyzed. 36 cases of ependymomas (51.4%) and 17 cases of other gliomas (68.0%) were classified as indistinguishable cases. Table 4 showed how well MRI differentiated schwannomas and ependymomas from meningiomas and other gliomas. Validity was lower in indistinguishable cases compared to overall cases. Kappa value indicating concordance with pathology also decreased in indistinguishable cases.

**Table 4:** Differentiation of schwannomas from meningiomas and ependymomas from other gliomas.

	Sensitivity^a^	Specificity^b^	Accuracy	Cohen's Kappa^c^
**Schwannoma vs. meningioma (IDEM)**				
Overall	0.876 (0.837, 0.915)	0.861 (0.796, 0.926)	0.872 (0.838, 0.905)	0.7 (0.622, 0.777)
Indistinguishable	0.675 (0.574, 0.775)	0.679 (0.506, 0.852)	0.676 (0.589, 0.763)	0.291 (0.118, 0.464)
**Ependymoma vs other glioma (IM)**				
Overall	0.9 (0.83, 0.97)	0.6 (0.408, 0.792)	0.821 (0.744, 0.898)	0.52 (0.321, 0.719)
Indistinguishable	0.833 (0.712, 0.955)	0.471 (0.233, 0.708)	0.717 (0.596, 0.838)	0.319 (0.046, 0.592)

Validities and their 95% confidence intervals (in parentheses).

^a^The sensitivity of schwannoma and ependymoma (specificity of meningioma and other glioma).

^b^The specificity of schwannoma and ependymoma (sensitivity of meningioma and other glioma).

^c^Cohen's Kappa coefficient between MRI report and pathologic report.

### Validity according to the period and neuroradiologist

**Figure 2:**
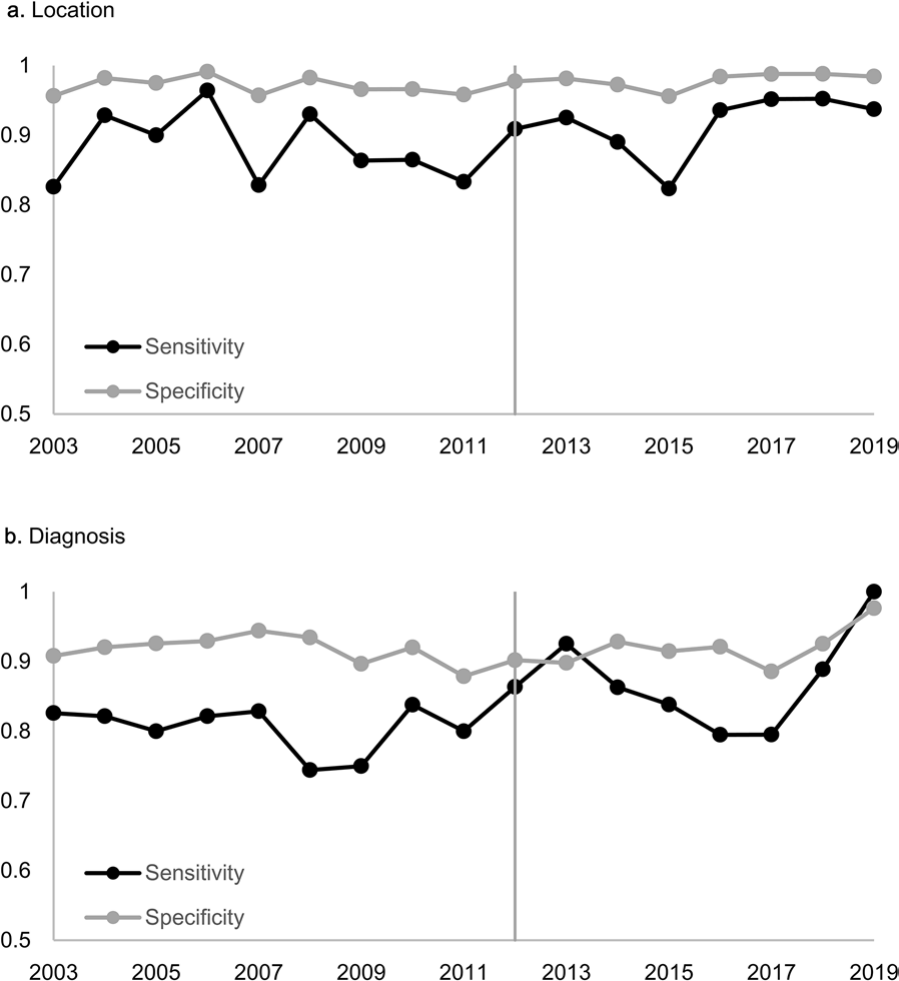
Validity according to the period of MRI examinations. (**a**) For the location, sensitivity and specificity showed no significant difference before and after 2012 (P = 0.943, 0.134). (**b**) For the diagnosis, there was no significant change in sensitivity and specificity ((P = 0.264, 0.581).

The first and second half were divided based on 2012, the middle of the study period. Additionally, 2012 was also the year when 3.0 T MRI began to be used in earnest at our institute. Figure 2 shows the change in sensitivity and specificity according to years before and after 2012. For the location, sensitivity and specificity showed no significant difference before and after 2012 (P = 0.943, 0.134). For the diagnosis, there was no significant change in sensitivity and specificity ((P = 0.264, 0.581). Table 5 shows the validity of the top 4 neuroradiologists for the number of cases. Overall sensitivity and specificity for location showed no significant difference between neuroradiologists. No. 2 tended to mention meningioma or ependymoma in other diseases. Thus, overall specificity for the diagnosis of No. 2 was significantly lower than those of the other three.

**Table 5:** Validity according to neuroradiologist.

Type	Neuroradiologist				p-value
	1	2	3	4	
**Location**					
Sensitivity	0.889 (0.839, 0.939)	0.932 (0.885, 0.978)	0.841 (0.771, 0.912)	0.921 (0.864, 0.978)	0.13
Specificity	0.972 (0.959, 0.985)	0.983 (0.971, 0.995)	0.960 (0.942, 0.979)	0.980 (0.966, 0.995)	0.16
**Diagnosis**					
Sensitivity	0.921 (0.873, 0.968)	0.969 (0.934, 1.004)	0.933 (0.879, 0.986)	0.924 (0.864, 0.984)	0.48
Specificity	0.893 (0.871, 0.914)	0.848 (0.819, 0.877)	0.922 (0.9, 0.945)	0.932 (0.876, 0.931)	< 0.001*

Asterisk indicates statistical significance.

## Discussion

Surgery planning and patient interviews are conducted based on MRIs. As well as diagnosis, information about tumor location is crucial to a surgeon. However, limited studies have been published with regards to the validity of MRI interpretations of primary spinal cord tumors. Only a few studies have focused on tumor location. This study was a large-scale study about tumor location and diagnosis. It included the largest number of cases compared to previous studies.

In our study, in contrast to previous results^2,9,10^, IDEM tumor was the most common (55.6%), and IM and ED tumors comprised 20.1% and 19.0%, respectively. Since this study focused on spinal canal lesions and excluded tumors originating from vertebrae, such as metastatic lesions, ED tumors were the least common.

Schwannomas and meningiomas are considered as some of the most common IDEM tumors. However, the single most common IDEM tumor is controversial^2,11–13^. Based on our study, schwannoma was the most common, while meningioma was less common. This result is consistent with a previous nationwide epidemiological study in Korea^14^. Ependymoma was the most common IM tumor, as in previous literature^1,4,5,9,10^. With metastatic lesions excluded, the most common ED tumor was schwannoma. The high frequency of ED cavernous malformations was interesting. However, considering the period of this study, the number is not particularly large. The frequency of IM cavernous malformation, which is known to be relatively common, was low. Since our institution treats this disease very conservatively^15,16^, it is thought that some IM cavernous malformations were omitted.

The sensitivity of MRI identification of tumor location was high. However, in cases with the tumor invading both intradural and extradural compartments simultaneously, the validity of MRI was reported to be poor. In addition, ten cases were misdiagnosed by IDEM, which was IM actually. 18 cases were interpreted as having an intradural part, which was purely an extradural tumor. It was difficult to predict the involvement of the intradural component. Hence, adequate consideration of intra- and extra-dural components of tumor would be needed for confusing cases^17^.

The validity of MRI in diagnosis showed high sensitivity and specificity. In particular, most tumors were diagnosed at the first and second impressions. In a similar study in the brain, the overall sensitivity and specificity were found to be 80%, which was similar to our results^18^. However, since this is an overall figure, prudence is required in interpretation. An important point in actual clinical practice is whether it is possible to discriminate even when the image characteristics overlap with other tumors. In other words, how well MRI can diagnose a confusing tumor would be important.

Hemangioblastoma, cavernous malformation, and benign cyst were diagnosed on MRI with high sensitivity and specificity (77.8 to 92.9%; 96.0 to 99.6%) since these tumors depict specific features on MRIs. Hemangioblastoma shows an intense enhancement with a large syrinx or flow void^11–13,19^. Cavernous malformation displays a dark rim on T2-weighted images. In addition, small-sized, eccentric axial location, minimal enhancement, and absence of edema are also significant MRI findings of cavernous malformation^20,21^. Benign cysts can be distinguished from a cystic change of other tumors by their location and enhancement pattern^22,23^.

Sensitivities for diagnosing schwannoma, meningioma, and ependymoma were in the range of 89.7 to 93.3%. Specificities were from 70.8 to 82.9%. Yan et al. reported that MRI yielded sensitivities of 88.4 to 95.7% for meningioma and 90.7 to 92.6% for schwannoma in a study on the validity of MRI in 764 brain tumors^18^. Those results are similar to our results. However, they also reported specificities of 94.8 to 97.0% and 99.9% for meningioma and schwannoma. In our study, the specificity for these tumors was low. This fact seems to be due to indistinguishable cases of schwannoma and meningioma in the spinal canal. Although meningiomas and schwannomas have typical imaging features—meningiomas have calcification and a dural tail, and schwannomas have a fluid signal and rim enhancement—their imaging features overlap in significant areas, such as the solid, round or oval, well-circumscribed contour of the lesion^1,2,7,10^. Some studies reported that up to 25% of schwannomas are indistinguishable from meningiomas^6,7^. In our study, approximately 30% of schwannoma and meningioma were classified as difficult to distinguish by MRI, and the accuracy and concordance rate with pathological diagnosis were quite low. Although MRI is useful for differentiating between spinal meningiomas and schwannomas, it is crucial to be aware that there are several cases that cannot be distinguished. Thus, close inspection of the intradural extra-arachnoid space would be needed to rule out meningioma. Then open the arachnoid membrane if there is no tumor in the extra-arachnoid space^17^.

The 76.0% specificity of ependymoma seems to be overestimated. Since most of the tumors were IDEM or ED tumors that did not require differentiation from ependymoma, true negatives were increased, and false positives were decreased. When evaluated with ependymomas and other gliomas, the specificity decreased to 60.0%. In addition, although the sensitivity of astrocytoma (76.9%) seems to be high, it was mostly diagnosed in the second impression. In half, ependymoma was considered first. These facts denote that neuroradiologists tend to diagnose glioma as ependymoma. Clear tumor margins, uniform enhancement, and the concentric feature can help distinguish ependymomas from other types of intramedullary tumors. However, differentiating ependymomas and astrocytomas may not be possible solely based on MRIs^24–26^.

In this study, most neurofibromas were diagnosed as a neurogenic tumor or benign nerve sheath tumor. Neurofibromas and schwannomas may look identical^1,2,4,10^. Hence, validation of neurofibroma seems to be meaningless.

Although the evolution of MRI over time has yielded high-quality radiologic information, it has not yet been fully utilized for the diagnosis of primary spinal cord tumors. There were no statistically significant changes over the period in both validity for tumor location and pathology. In addition, the validity varies according to the neuroradiologists. These facts suggest that a system that can meticulously analyze currently available radiologic information is needed rather than hardware development.

The present study is subject to several limitations. First, the study was retrospective. However, bias was minimized since the data had been collected prospectively. In addition, considering the rarity of primary spinal cord tumors, it would not be easy to perform a prospective study with a better design than this one. Second, we did not re-interpret MRI scans, which may have influenced the sensitivity and specificity of MRIs. Nevertheless, this point can be an advantage since it reflects routine practice. Third, this study was a review of 14 years of data conducted at a single center, which is a tertiary referral hospital. Hence, it would be a challenge to generalize the results of this study. In addition, neuroradiologists at this hospital are experts who have seen many primary spinal cord tumors. Thus, it would be hard to generalize the present results as reflecting the average neuroradiologists' performance.

Nevertheless, this study reveals that there are still many things to improve. For example, there is considerable variability in the specificity and sensitivity of diagnoses for various lesions. More importantly, the sensitivity of rare tumors is low. Possibly this is because these rare tumors have no imaging feature distinguishing them from other tumors, and the number of cases experienced by neuroradiologists may also be different. Location is also difficult to track if it spans multiple spaces. Even in rare cases, it is important to increase sensitivity to maintain a uniform validity. In addition, technological advances are not fully translated into a diagnosis of primary spinal cord tumors. Thus, it is necessary to find a way to set up a system that can analyze advanced MRI information, such as establishing radiologic diagnostic criteria or introducing artificial intelligence.

## Conclusion

MRI is the preoperative modality of choice in the evaluation of primary spinal cord tumors. The validities of MRIs for diagnosing primary spinal cord tumors and predicting their location are generally satisfactory. However, the validity differs among tumor types; hemangioblastoma, cavernous malformation, and benign cyst are more likely to be diagnosed correctly. Schwannoma, meningioma, and ependymomas are also acceptable in general cases, while poor in confusing cases. Rare tumors are poorly diagnosed. Hence, it is essential to be aware that there are tumors that cannot be distinguished from others by MRI and that MRI diagnosis still needs some improvement.

## Supplementary Information

Supplementary Tables.
