# Supplementary material for: Validity of magnetic resonance imaging (MRI) in the primary spinal cord tumors in routine clinical setting
Source: Sci Rep. 2022 Jun 16;12:10151. doi: 10.1038/s41598-022-13881-z (PMC9203586; doi:10.1038/s41598-022-13881-z)
Supplement: Supplementary file 1 — Supplementary Tables. [file 41598_2022_13881_MOESM1_ESM.docx]

**TABLE S1. The validity for the location according to spine level.**

| Location | | Cervical | Thoracic | Thoracolumbar | Lumbar | p-value |
| --- | --- | --- | --- | --- | --- | --- |
| IDEM | Sensitivity | 0.861^a^  (0.783, 0.939) | 0.975  (0.95, 0.999) | 0.929  (0.859, 0.998) | 0.927  (0.887, 0.967) | **0.01^*^** |
|  | Specificity | 0.939  (0.902, 0.976) | 0.928  (0.875, 0.98) | 0.825  (0.702, 0.948) | 0.921  (0.852, 0.989) | 0.121 |
| IM | Sensitivity | 0.988  (0.963, 1.012) | 0.898  (0.819, 0.978) | 0.846^b^  (0.698, 0.995) |  | **0.014^*^** |
|  | Specificity | 0.957  (0.926, 0.989) | 0.974  (0.952, 0.997) | 0.987  (0.961, 1.013) |  | 0.458 |
| ED | Sensitivity | 0.852  (0.761, 0.944) | 0.909  (0.806, 1.013) | 0.867  (0.672, 1.062) | 0.922  (0.845, 0.998) | 0.616 |
|  | Specificity | 0.978  (0.957, 1) | 0.991  (0.978, 1.004) | 0.963  (0.921, 1.005) | 0.966  (0.939, 0.993) | 0.236 |
| ID&ED | Sensitivity | 0.632  (0.393, 0.87) | 0.4  (-0.28, 1.08) | 0.333  (-1.101, 1.768) | 0.5  (-0.075, 1.075) | 0.673 |
|  | Specificity | 0.955^c^  (0.928, 0.983) | 0.996  (0.988, 1.004) | 0.978  (0.948, 1.009) | 0.991  (0.978, 1.004) | **0.008^*^** |
| IM&EM | Sensitivity | 0.375  (-0.058, 0.808) |  | 0  (0, 0) |  | 0.533 |
|  | Specificity | 0.991  (0.98, 1.003) | 0.996  (0.988, 1.004) | 1  (1, 1) |  | 0.77 |
| Total | Sensitivity | 0.864^d^  (0.821, 0.908) | 0.937  (0.907, 0.967) | 0.865  (0.795, 0.934) | 0.912  (0.875, 0.949) | **0.029^*^** |
|  | Specificity | 0.966^d^  (0.955, 0.977) | 0.984  (0.977, 0.992) | 0.966  (0.948, 0.984) | 0.978  (0.968, 0.988) | **0.04^*^** |

IDEM = Inradural extra, IM = Intramedullary, ED = Extradural, ID&ED = both intradural and extradural, IM&EM = both intramedullary and extramedullary

Chi-square test or Fisher's exact test was performed to determine whether the validity for location differs according to the spine level.

^a^Post hoc analysis revealed the sensitivity of cervical was lower than thoracolumbar (P=0.006).

^b^The sensitivity of thoracolumbar was lower than cervical (P=0.031).

^c^The specificity of cervical was lower than thoracic (P=0.007).

^d^The sensitivity and specificity of cervical were lower than thoracic (P=0.034, 0.046).

Asterisk indicates statistical significance.

**TABLE S2. Distribution of tumors according to spine level.**

| Tumor Type | Cervical | Thoracic | Thoracolumbar | Lumbar | p-value |
| --- | --- | --- | --- | --- | --- |
| Schwannoma | 95 (39.1%)^a^ | 95 (37.4%)^a^ | 60 (62.5%) | 166 (73.1%) | **<0.001^*^** |
| Meningioma | 29 (11.9%) | 72 (28.4%)^b^ | 6 (6.3%) | 7 (3.1%) ^b^ | **<0.001^*^** |
| Ependymoma | 45 (18.5%)^c^ | 22 (8.7%) | 7 (7.3%) | 13 (5.7%) | **<0.001^*^** |
| Hemagioblastoma | 15 (6.2%) | 9 (3.5%) | 1 (1.0%) | 3 (1.3%)^d^ | **0.013^*^** |
| Cavernous malformation | 9 (3.7%) | 13 (5.1%) | 1 (1.0%) | 4 (1.8%) | 0.09 |
| Benign cyst | 5 (2.1%) | 7 (2.8%) | 1 (1.0%) | 9 (4.0%) | 0.402 |
| Neurofibroma | 10 (4.1%) | 4 (1.6%) | 2 (2.1%) | 1 (0.4%)^e^ | **0.035^*^** |
| Astrocytoma | 6 (2.5%) | 4 (1.6%) | 3 (3.1%) |  | 0.629 |
| Diffuse midline glioma | 3 (1.2%) | 3 (1.2%) | 4 (4.2%) |  | 0.192 |
| Total | 243 | 254 | 96 | 227 | **<0.001^*^** |

% means the proportion of tumors at the spine level.

Chi-square test or Fisher's exact test was performed to determine whether the distribution of tumors differs according to the spine level.

^a^Post hoc analysis revealed the proportion of schwannoma at cervical and thoracic were lower than at thoracolumbar and lumbar (P<0.001).

^b^The proportion of meningioma at thoracic was higher than the other levels (P<0.001), and the proportion at lumbar was lower than at cervical (P<0.001).

^c^The proportion of ependymoma at cervical was higher than the other levels (P<0.001).

^d^The proportion of hemangioblastoma at lumbar was lower than at cervical (P=0.012)

^e^The proportion of neurofibroma at lumbar was lower than cervical (P=0.020).

Asterisk indicates statistical significance.

**TABLE S3. The validity for the diagnosis according to spine level.**

| Diagnosis | | Cervical | Thoracic | Thoracolumbar | Lumbar | p-value |
| --- | --- | --- | --- | --- | --- | --- |
| Schwannoma | Sensitivity | 0.926  (0.873, 0.98) | 0.905  (0.845, 0.965) | 0.917  (0.845, 0.989) | 0.958   (0.927, 0.989) | 0.374 |
|  | Specificity | 0.818  (0.755, 0.881) | 0.717  (0.646, 0.788) | 0.583^a^  (0.414, 0.753) | 0.492^a^  (0.363, 0.621) | **<0.001^*^** |
| Meningioma | Sensitivity | 0.793  (0.636, 0.95) | 0.944  (0.89, 0.999) | 1  (1, 1) | 0.857  (0.508, 1.207) | 0.095 |
|  | Specificity | 0.879  (0.834, 0.923) | 0.775  (0.713, 0.836) | 0.811  (0.729, 0.894) | 0.832  (0.782, 0.882) | 0.053 |
| Ependymoma | Sensitivity | 0.978  (0.933, 1.023) | 0.818  (0.643, 0.993) | 0.714  (0.263, 1.166) | 0.846  (0.619, 1.073) | 0.057 |
|  | Specificity | 0.854  (0.804, 0.903) | 0.853  (0.808, 0.899) | 0.596^b^  (0.492, 0.699) | 0.64^b^  (0.575, 0.705) | **<0.001^*^** |
| Hemagioblastoma | Sensitivity | 0.933  (0.79, 1.076) | 1  (1, 1) | 1  (1, 1) | 0.667  (-0.768, 2.101) | 0.365 |
|  | Specificity | 0.943  (0.913, 0.973) | 0.955  (0.929, 0.981) | 0.989  (0.969, 1.01) | 0.982  (0.965, 1) | 0.065 |
| Cavernous malformation | Sensitivity | 0.778  (0.439, 1.117) | 0.846  (0.619, 1.073) | 1  (1, 1) | 0.5  (-0.419, 1.419) | 0.517 |
|  | Specificity | 0.94  (0.91, 0.971) | 0.979  (0.961, 0.997) | 0.947  (0.902, 0.993) | 0.964  (0.94, 0.989) | 0.16 |
| Benign cyst | Sensitivity | 1  (1, 1) | 0.857  (0.508, 1.207) | 1  (1, 1) | 0.778  (0.439, 1.117) | 0.795 |
|  | Specificity | 1  (1, 1) | 1  (1, 1) | 1  (1, 1) | 0.986^c^  (0.971, 1.002) | **0.046^*^** |
| Neurofibroma | Sensitivity | 0.2  (-0.102, 0.502) | 0.5  (-0.419, 1.419) | 0.5  (-5.853, 6.853) | 0  (0, 0) | 0.593 |
|  | Specificity | 0.931^c^  (0.899, 0.964) | 0.976  (0.957, 0.995) | 0.947  (0.901, 0.993) | 0.978  (0.959, 0.997) | **0.029^*^** |
| Astrocytoma | Sensitivity | 0.833  (0.405, 1.262) | 1  (1, 1) | 0.333  (-1.101, 1.768) |  | 0.185 |
|  | Specificity | 0.861  (0.816, 0.905) | 0.912  (0.877, 0.947) | 0.936  (0.886, 0.987) |  | 0.067 |
| Diffuse midline glioma | Sensitivity | 0.333  (-1.101, 1.768) | 0.333  (-1.101, 1.768) | 0.25  (-0.546, 1.046) |  | 0.972 |
|  | Specificity | 1  (1, 1) | 0.996  (0.988, 1.004) | 1  (1, 1) |  | 0.517 |
| Total | Sensitivity | 0.794  (0.743, 0.845) | 0.846  (0.802, 0.891) | 0.771  (0.685, 0.856) | 0.881^c^  (0.839, 0.923) | **0.024^*^** |
|  | Specificity | 0.92  (0.908, 0.932) | 0.92  (0.908, 0.932) | 0.891  (0.869, 0.913) | 0.909  (0.896, 0.922) | 0.059 |

Chi-square test or Fisher's exact test was performed to determine whether the validity for diagnosis differs according to the spine level.

^a^Post hoc analysis revealed the specificity of thoracolumbar was lower than cervical (P=0.24) and lumbar was lower than cervical and thoracic (P<0.001).

^b^The specificities of thoracolumbar and lumbar were lower than cervical and thoracic (P<0.001).

^c^No statistically significant difference between spine levels.

Asterisk indicates statistical significance.
